# The gut microbiota as a modulator of innate immunity during melioidosis

**DOI:** 10.1371/journal.pntd.0005548

**Published:** 2017-04-19

**Authors:** Jacqueline M. Lankelma, Emma Birnie, Tassili A. F. Weehuizen, Brendon P. Scicluna, Clara Belzer, Riekelt H. Houtkooper, Joris J. T. H. Roelofs, Alex F. de Vos, Tom van der Poll, Andries E. Budding, W. Joost Wiersinga

**Affiliations:** 1 Center for Experimental and Molecular Medicine, Academic Medical Center, University of Amsterdam, Amsterdam, The Netherlands; 2 Department of Clinical Epidemiology, Biostatistics and Bioinformatics, Academic Medical Center, University of Amsterdam, Amsterdam, the Netherlands; 3 Laboratory of Microbiology, Wageningen University, Wageningen, The Netherlands; 4 Laboratory Genetic Metabolic Diseases, Academic Medical Center, University of Amsterdam, Amsterdam, The Netherlands; 5 Department of Pathology, Academic Medical Center, University of Amsterdam, Amsterdam, The Netherlands; 6 Department of Internal Medicine, Division of Infectious Diseases, Academic Medical Center, Amsterdam, The Netherlands; 7 Department of Medical Microbiology, Vrije Universiteit, Amsterdam, The Netherlands; University of Tennessee, UNITED STATES

## Abstract

**Background:**

Melioidosis, caused by the Gram-negative bacterium *Burkholderia pseudomallei*, is an emerging cause of pneumonia-derived sepsis in the tropics. The gut microbiota supports local mucosal immunity and is increasingly recognized as a protective mediator in host defenses against systemic infection. Here, we aimed to characterize the composition and function of the intestinal microbiota during experimental melioidosis.

**Methodology/Principal findings:**

C57BL/6 mice were infected intranasally with *B*. *pseudomallei* and sacrificed at different time points to assess bacterial loads and inflammation. In selected experiments, the gut microbiota was disrupted with broad-spectrum antibiotics prior to inoculation. Fecal bacterial composition was analyzed by means of IS-pro, a 16S-23S interspacer region-based profiling method. A marked shift in fecal bacterial composition was seen in all mice during systemic *B*. *pseudomallei* infection with a strong increase in Proteobacteria and decrease in Actinobacteria, with an increase in bacterial diversity. We found enhanced early dissemination of *B*. *pseudomallei* and systemic inflammation during experimental melioidosis in microbiota-disrupted mice compared with controls. Whole-genome transcriptional profiling of the lung identified several genes that were differentially expressed between mice with a normal or disrupted intestinal microbiota. Genes involved in acute phase signaling, including macrophage-related signaling pathways were significantly elevated in microbiota disrupted mice. Compared with controls, alveolar macrophages derived from antibiotic pretreated mice showed a diminished capacity to phagocytose *B*. *pseudomallei*. This might in part explain the observed protective effect of the gut microbiota in the host defense against pneumonia-derived melioidosis.

**Conclusions/Significance:**

Taken together, these data identify the gut microbiota as a potential modulator of innate immunity during *B*. *pseudomallei* infection.

## Author summary

Melioidosis is a common cause of community-acquired pneumonia and sepsis in Asia. The causative agent, *Burkholderia pseudomallei*, is listed as a potential bioterror weapon. The intestinal microbiota has been suggested to be a modulator of innate immune defenses against bacterial infections. Here we investigated in mice whether the intestinal microbiota affects the clinical course of melioidosis and vice versa. The composition of the gut microbiota changed strongly during melioidosis. Mice with a disrupted gut microbiota showed increased bacterial dissemination when compared with controls following intranasal infection with *B*. *pseudomallei*, indicating that the intestinal microbiota acts as a protective factor in host defense against melioidosis. Macrophages in microbiota-disrupted mice showed a diminished capacity to phagocytose *B*. *pseudomallei*. Further research is needed to explore whether this knowledge could be used to the advantage of patients.

## Introduction

Melioidosis is a frequent cause of community-acquired sepsis in Southeast Asia and northern Australia [1, 2]. Pneumonia is the presenting symptom in most adult patients [3] and results in a rapidly progressive illness with a high mortality up to 40% [1, 3]. The disease is caused by *Burkholderia pseudomallei*, a facultative intracellular Gram-negative bacterium that is commonly found in the soil from countries located between 20° north latitude and 20° south latitude [1, 2, 4]. Due to its high lethality, poor sensitivity to antibiotics, wide availability and easy dissemination, it has been classified as a Tier 1 biological threat agent. The global burden of melioidosis is probably much larger than previously anticipated: it was recently estimated that each year 165,000 (95% credible interval 68,000–412,000) people suffer from this debilitating disease resulting in 89,000 (36,000–227,000) fatalities [5]. Melioidosis is probably underreported in as many as 45 countries due to a lack of adequate diagnostic facilities [5]. In the near future, the management of patients may be compromised by emergence of resistance due to increased use of antibiotics in endemic regions [6]. Thus, there is an urgent need to better understand the pathogenesis of melioidosis.

The intestinal microbiota not only provides direct colonization resistance against invading pathogens, but is increasingly recognized as an important modulator of systemic immunity [7–9]. It was first suggested by Clarke and colleagues that bacterial cell wall components such as peptidoglycan are translocated into the bloodstream and at distant sites ‘prime’ immune effector cells [10]. This way, the effectiveness of bone-marrow derived neutrophils in killing pathogens such as *Streptococcus pneumoniae* and *Staphylococcus aureus* is increased [10]. Subsequent studies could demonstrate a protective effect of a healthy microbiota in a variety of in vivo murine models of infection: *S*. *aureus*, *Pseudomonas aeruginosa* or *Klebsiella pneumoniae* pneumonia [11–13] and *Listeria monocytogenes* or *Escherichia coli* induced sepsis [14, 15]. In line, we recently demonstrated that the gut microbiota plays a protective role in pathogenesis of pneumococcal pneumonia by enhancing primary alveolar macrophage function [16].

To the best of our knowledge, the role of the gut microbiota in the host defense against melioidosis has never been investigated. The importance of this subject is underscored by the notion that melioidosis has a notoriously protracted course for which cure can only be achieved through long-term antibiotic therapy. The minimum of two weeks of intravenous antibiotics followed by three months of oral antibiotics [1, 2] will have a profound effect on the microbiota. We hypothesized that a healthy microbiota supports the host defense against *B*. *pseudomallei* infection. In order to address this question, we made use of our well-established murine model of pneumonia-derived melioidosis [17, 18] and in selected experiments disrupted the gut microbiota with oral antibiotics before infection following a standard protocol [10, 16]. We show that the intestinal microbiota changes significantly during melioidosis, independent of antibiotic treatment. Secondly, we show that antibiotic disruption of the intestinal microbiota is associated with a less effective innate immune defense against experimental *B*. *pseudomallei* infection.

## Results

### Profound changes in gut microbiota composition during murine pneumonia-derived melioidosis

**Fig 1:**
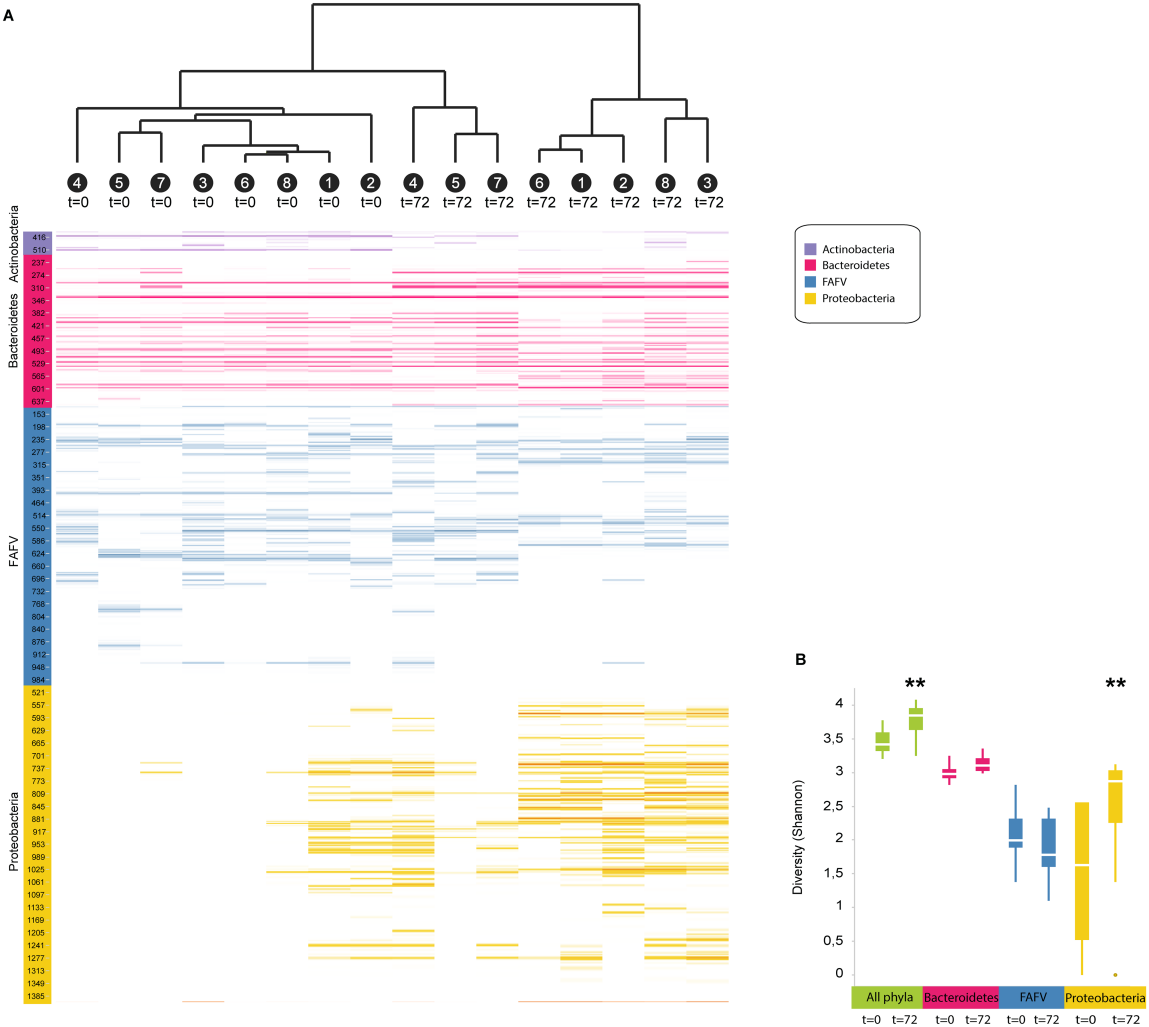
Profound changes in fecal microbiota composition during melioidosis. Fecal pellets were sampled from eight mice before (t = 0) they were infected intranasally with 150 CFU of *B*. *pseudomallei* and 72 hours after (t = 72). Microbial composition was analysed by IS-pro, using the number of nucleotides between the genes for ribosomal subunit 16 and 23 in the DNA (interspacer region) of the bacterium as a unique classification characteristic. (A) Clustering analysis, by unweighted pair group method with arithmetic mean (UPGMA) on cosine distances, shows the similarity of samples; individual mice are indicated by a number. Colors represent the most important bacterial phyla (purple, Actinobacteria; red, Bacteroidetes; blue, Firmicutes, Actinobacteria, Fusobacteria, and Verrucomicrobia (FAFV); yellow, Proteobacteria). Length of the interspacer regions in basepairs is indicated on the y-axis; lines indicate the presence of PCR products. Color intensity increases with the presence of PCR product. (B) Diversity of microbial communities before and 72 hours after induction of melioidosis, expressed as Shannon index (green: total bacteria; red: Bacteroidetes; Blue: Firmicutes, Actinobacteria, Fusobacteria, and Verrucomicrobia (FAFV); yellow: Proteobacteria). Data are presented as box- and whisker plots showing the smallest observation, lower quartile, median, upper quartile and largest observation. ** p<0.01 pre- versus post-infection.

To first obtain insight into the composition of the intestinal microbiota during melioidosis, we inoculated mice intranasally with live *B*. *pseudomallei* to induce pneumonia-derived melioidosis and collected fecal pellets at baseline (t = 0) and after 72 hours, when all mice had symptoms of systemic infection. The gut microbiota was analysed by IS-pro technique, using the number of nucleotides between the genes for the 16S and 23S ribosomal subunits in bacterial DNA as a unique classification characteristic [19, 20]. Infection with *B*. *pseudomallei* was associated with profound changes in the composition of the intestinal microbiota (Fig 1). Clustering analysis, by unweighted pair group method with arithmetic mean (UPGMA) on cosine distances of all samples, resulted in separation of all pre- and post-infection samples (t = 0 vs t = 72), indicating that pre- and post-infection samples from each mouse were highly dissimilar. In all mice, a strong increase in Proteobacteria was seen as well as a decrease in Actinobacteria. The composition of Bacteroides and Firmicutes also changed in strikingly similar patterns. Of note, *B*. *pseudomallei* was not detected in any fecal sample. Total microbial diversity was significantly increased (p = 0.006), mostly due to increased diversity of Bacteroides (p = 0.055) and Proteobacteria (p = 0.007) (Fig 1).

### Antibiotic gut microbiota disruption is associated with increased growth and dissemination of *B*. *pseudomallei*

**Fig 2:**
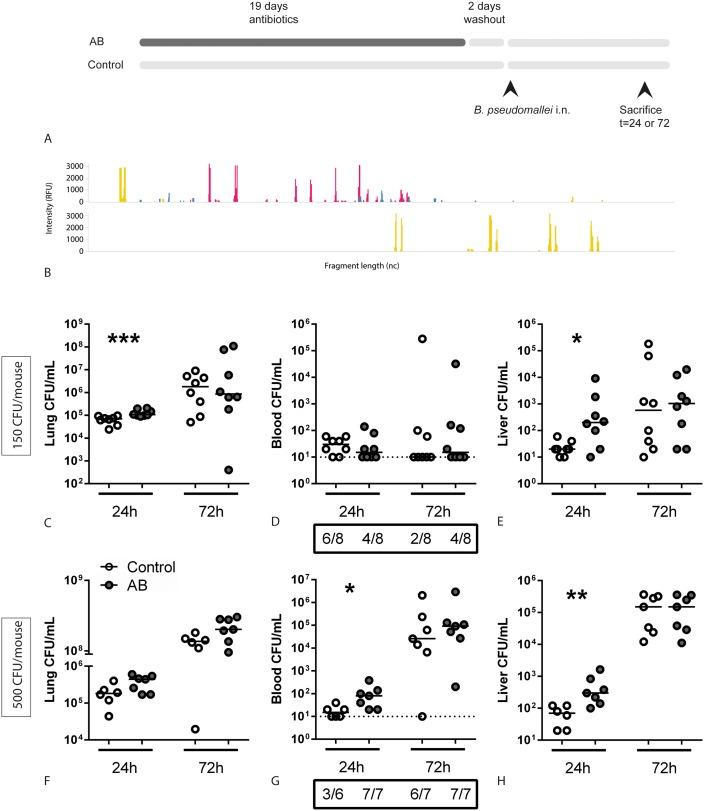
Antibiotic pre-treated mice show increased growth and dissemination of *B*. *pseudomallei* during experimental melioidosis. (A) Study design. (B) Before infection, the fecal microbiota of control- and antibiotic treated mice was analysed by IS-pro. Colors represent the most important bacterial phyla (purple, Actinobacteria; red, Bacteroidetes; blue, Firmicutes, Actinobacteria, Fusobacteria, and Verrucomicrobia (FAFV); yellow, Proteobacteria). Length of the interspacer regions in basepairs is indicated on the x-axis; height of the peaks indicates the presence of PCR products. Samples are pooled from eight mice per group; representative of two experiments. Control and antibiotic pre-treated mice were inoculated intranasally with 150 CFU (C-E) or 500 CFU (F-H) *B*. *pseudomallei* and sacrificed at the indicated time points. Bacterial loads in lung homogenate (C, F), blood (D, G) and liver homogenate (E, H) are depicted as scatter dot plots with a line at the median. Numbers in the boxes below (D) and (G) indicate the number of positive blood cultures for the total number of mice. White dots represent control mice, grey dots antibiotic treated mice. N = 6–8 mice per group. * p<0.05, ** p<0.01, *** p<0.001 control versus antibiotic treated.

To investigate whether gut microbiota composition impacts on host defense during melioidosis, we pre-treated mice with broad-spectrum antibiotics in drinking water in order disrupt the intestinal microbiota (Fig 2A) [10, 16]. We then inoculated mice intranasally with live *B*. *pseudomallei* (150 colony forming units (CFU), LD50) [17, 18] and sacrificed them after 24 or 72 hours. The antibiotic treatment caused dramatic changes in the intestinal microbiota compared to untreated control mice, with a marked reduction in the number of species (Fig 2B). Relative to control mice, antibiotic pre-treated mice displayed significantly increased bacterial loads in lung and liver 24 hours after infection (Fig 2C–2E). Bacterial loads in blood and broncho-alveolar lavage fluid (BALF) were not affected (Fig 2D and S1 Fig). To determine whether the effect of gut microbiota disruption on bacterial growth was dependent on the infectious dose, we next infected mice with 500 CFU *B*. *pseudomallei* (LD100). Using this higher infectious dose, the observed increase in bacterial dissemination in antibiotic treated mice was also present at the early time-point following infection (Fig 2F–2H). In addition, we tested whether the observed effects were specific for this combination of antibiotics by performing the same experiment, using only metronidazole and ampicillin in drinking water (S2 Fig). Similar to the previous experiments, we again observed increased bacterial loads after 24 hours in lungs of antibiotic pre-treated mice compared to controls—indicating that the intestinal bacteria targeted by these two antibiotics are involved in the observed effects.

### Gut microbiota disruption is associated with increased proinflammatory cytokine release during experimental melioidosis

Having found an inoculum-dependent effect of antibiotic pre-treatment on bacterial growth both at the primary site of infection and at distant sites, we next studied the impact of antibiotic pre-treatment on local and systemic cytokine release in mice infected with 500 CFU *B*. *pseudomallei*. In lung homogenate, cytokine levels were similar between groups at all time-points (Table 1). Plasma levels of tumor necrosis factor (TNF)-α and interferon (IFN)-γ however were significantly increased in mice with a disrupted gut microbiota, 72 hours after infection (Table 1).

**Table 1:** Cytokine- and chemokine levels in lung homogenate and plasma.

(pg/mL)	24h		72h	
	Control (n = 6)	AB (n = 7)	Control (n = 7)	AB (n = 7)
	**Lung homogenate**			
**TNFα**	5591 [4645–5757]	5486 [5174–7869]	20045 [15888–21589]	14716 [13987–18619]
**IL-1β**	558 [292–795]	455 [359–690]	6428 [3530–8950]	6938 [4744–7526]
**IL-6**	22238 [8984–13448]	11482 [10748–13201]	36048 [31819–38015]	44096 [36123–56812]
**IFNγ**	484 [272–614]	466 [419–506]	399 [297–507]	455 [321–607]
**CXCL1**	19128 [14129–24154]	17066 [12866–21757]	79927 [72121–90611]	97584 [65108–112425]
	**Plasma**			
**TNFα**	10 [3–26]	27 [3–31]	225 [27–1183]	3566 [564–5000] *
**IFNγ**	16 [8–27]	27 [23–31]	64 [12–199]	1030 [171–2566] *
**IL-6**	135 [44–234]	285 [243–472] *	14091 [2055–21061]	25376 [11879–71534]
**CCL2**	220 [92–284]	213 [168–290]	569 [31–3820]	3238 [1848–4409]

Cytokine and chemokine levels in lung homogenate and plasma from mice inoculated with 500 CFU *B*. *pseudomallei*, sacrificed 24 or 72 hours post infection. Values are in pg/mL and presented as median [interquartile range].

* p<0.05 control versus antibiotic treated.

### Limited impact of antibiotic induced gut microbiota disruption on survival and organ injury

**Fig 3:**
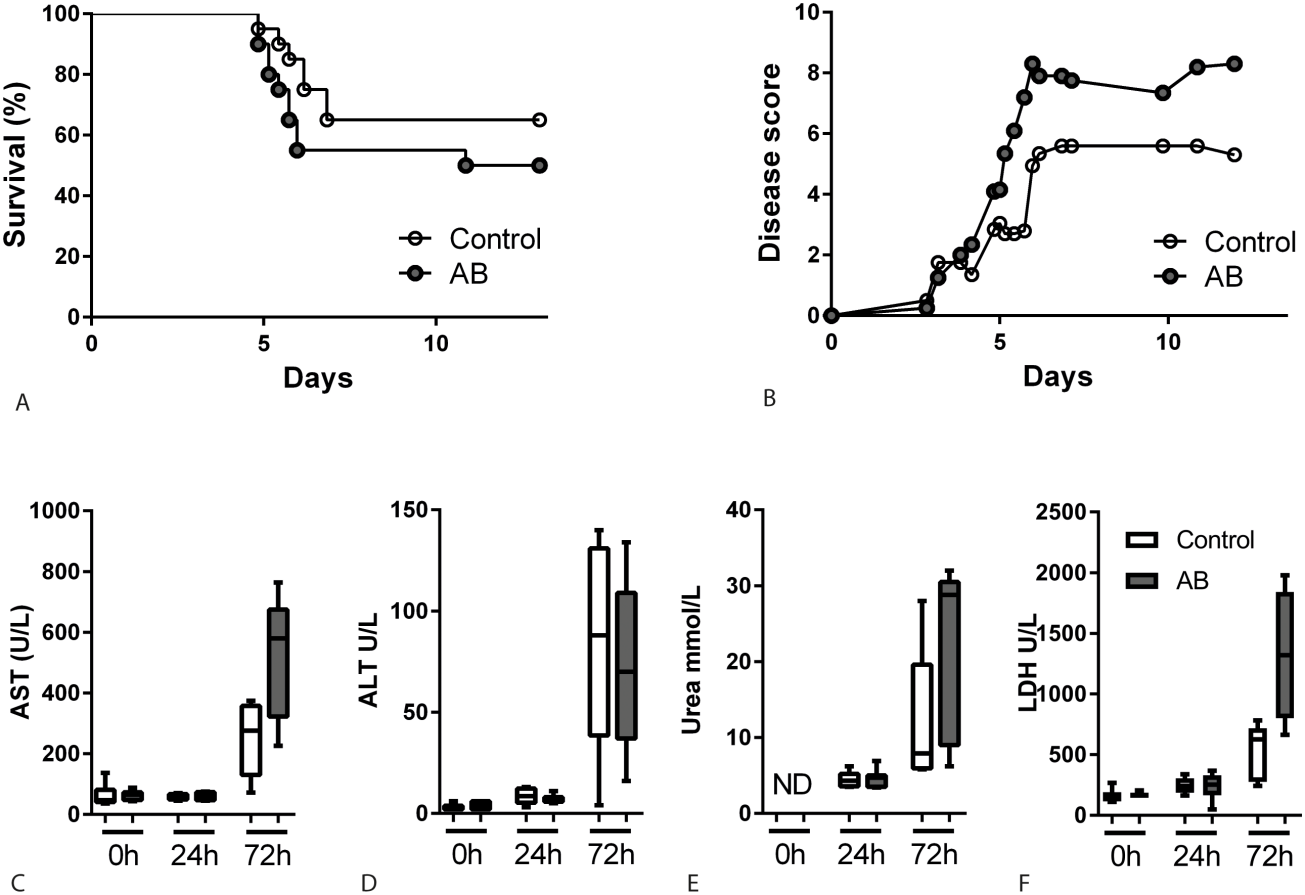
Limited effect of antibiotic induced gut microbiota disruption on survival and organ damage. Survival (A) and clinical observation score (B) of control (white dots) and antibiotic treated mice (grey dots) after intranasal inoculation with 150 CFU *B*. *pseudomallei* (n = 20 mice per group, depicted is the mean). No statistically significant differences were detected. Aspartate aminotranspherase (AST, C), alanine aminotranspherase (ALT, D), urea (E) and lactate dehydrogenase (LDH, F) were measured in plasma after inoculation with 500 CFU *B*. *pseudomallei* as markers for liver-, renal- and general damage. Data are presented as box- and whisker plots showing the smallest observation, lower quartile, median, upper quartile and largest observation. White bars represent control mice, grey bars antibiotic treated mice. N = 5–6 samples per group. ND = not detectable.

To evaluate whether the above findings would lead to impaired survival in the experimental group, we followed groups of 20 mice for 14 days after intranasal inoculation with 150 CFU *B*. *pseudomallei*. The lower dose was chosen since this LD50 [17, 18] would allow to demonstrate a potential detrimental effect of gut microbiota depletion. Mice in the antibiotic pre-treated group showed a trend toward increased mortality but this did not reach statistical significance (Fig 3A). Likewise, a clinical observation score reflected a trend towards increased morbidity in the experimental group (Fig 3B). The marked organ injury in this model of melioidosis is reflected by elevated plasma markers of hepatocellular damage (aspartate aminotranspherase, AST and alanine aminotranspherase, ALT), renal failure (urea) and general cellular damage (lactate dehydrogenase, LDH), especially shortly before mortality occurs [17, 18] (Fig 3C–3F). However, in line with survival, no significant differences in these parameters were observed, indicating a limited influence of gut microbiota disruption on the extent of organ damage.

### Intestinal microbiota does not influence neutrophils during melioidosis

**Fig 4:**
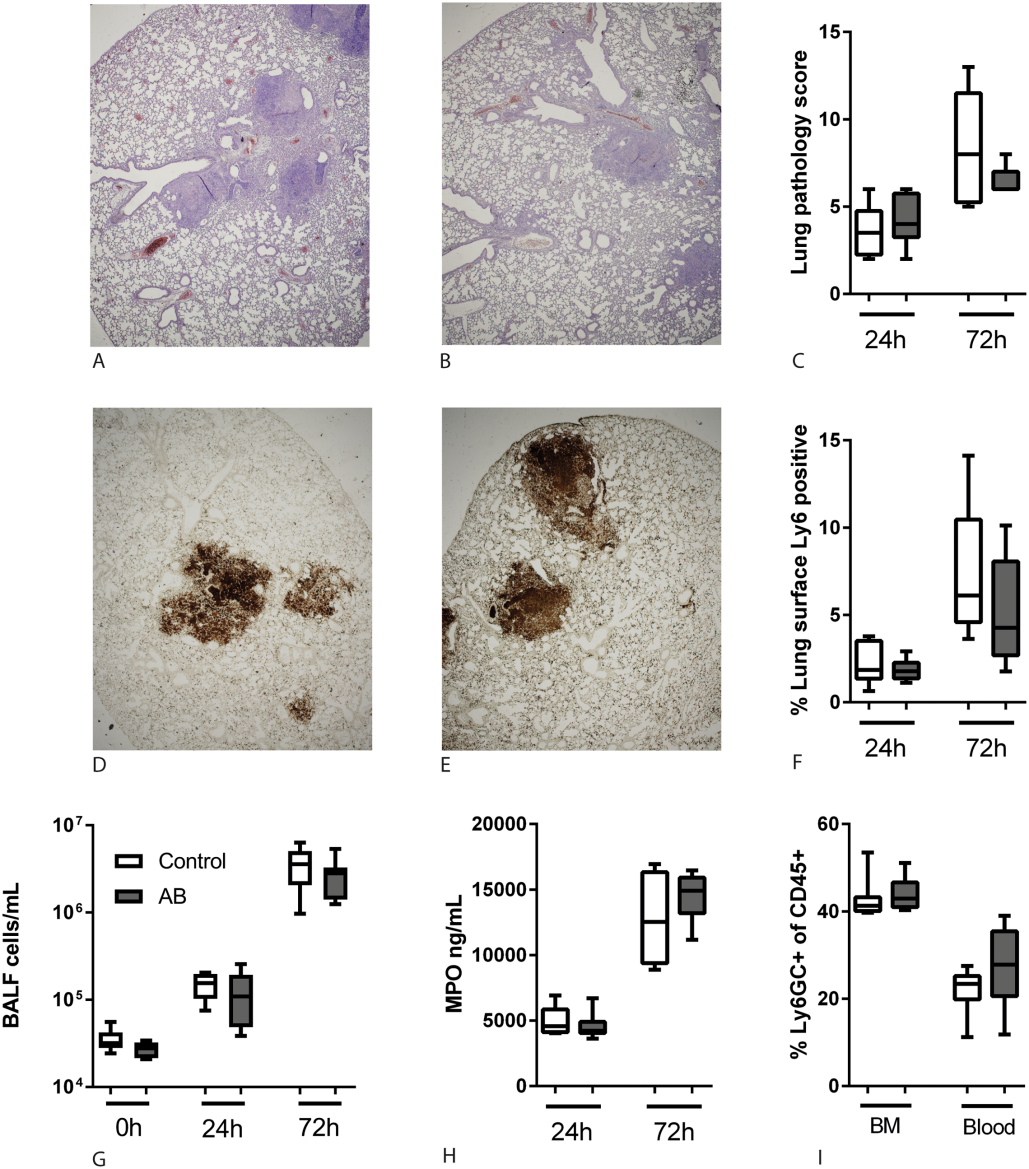
Antibiotic microbiota disruption does not affect neutrophil influx. Lungs were obtained at the indicated time points after intranasal inoculation with 500 CFU *B*. *pseudomallei*. Paraffin-embedded lung tissue sections were stained with haematoxylin/eosin to score different parameters for pathology (A-B, 2x magnification, representative images). The combined score, given by a blinded pathologist, was not different between the two groups (C). Sections from the same samples were stained for Ly-6GC as a neutrophil marker (representative microphotographs, 2x magnification) (D-E). The percentage Ly-6GC-positive surface of the total lung surface was calculated using ImageJ (F). The number of cells per mL BALF was counted using a Coulter counter (G). Myeloperoxidase was quantified in lung homogenates as a measure for neutrophil degranulation (H). Bone marrow and blood was obtained from naïve control and antibiotic pre-treated mice and using FACS analysis the percentage of Ly6GC+, CD11b+ cells within the viable CD45+ population was determined (I). Data are presented as box- and whisker plots showing the smallest observation, lower quartile, median, upper quartile and largest observation. White bars represent control mice, grey bars antibiotic treated mice. No statistically significant differences were found.

As the microbiota has been reported to be an important regulator of neutrophil homeostasis [10, 14, 15, 21] and neutrophils play an essential role in the host defense against melioidosis [22, 23], we hypothesized that these might play a role in the observed differences. 72 hours after infection, all mice showed extensive lung infiltrates characterized by neutrophil influx, necrosis, bronchitis, endothelialitis and oedema (Fig 4A and 4B). However, when we analysed HE-stained lung tissue sections using a semi-quantitative pathology scoring system, no differences were found between control and antibiotic pre-treated mice (Fig 4C). Quantification of a Ly-6GC staining demonstrated a similar pulmonary influx of neutrophils in both groups (Fig 4D–4F). In line, a similar pulmonary influx of cells was observed in BALF during melioidosis (Fig 4G). Equal neutrophil degranulation was confirmed by lung myeloperoxidase levels in control and antibiotic treated mice after infection with *B*. *pseudomallei* (Fig 4H). Similar results were obtained for mice inoculated with 150 or 500 CFU; only the latter are shown. Lastly, since a healthy microbiota is proposed to stimulate granulopoiesis [10, 14, 15, 21], we studied neutrophil numbers in bone marrow and blood of naïve control and antibiotic treated mice; however, we did not find any differences (Fig 4I).

### Altered lung transcriptome and impaired phagocytosis of *B*. *pseudomallei* by alveolar macrophages in gut microbiota disrupted mice

**Fig 5:**
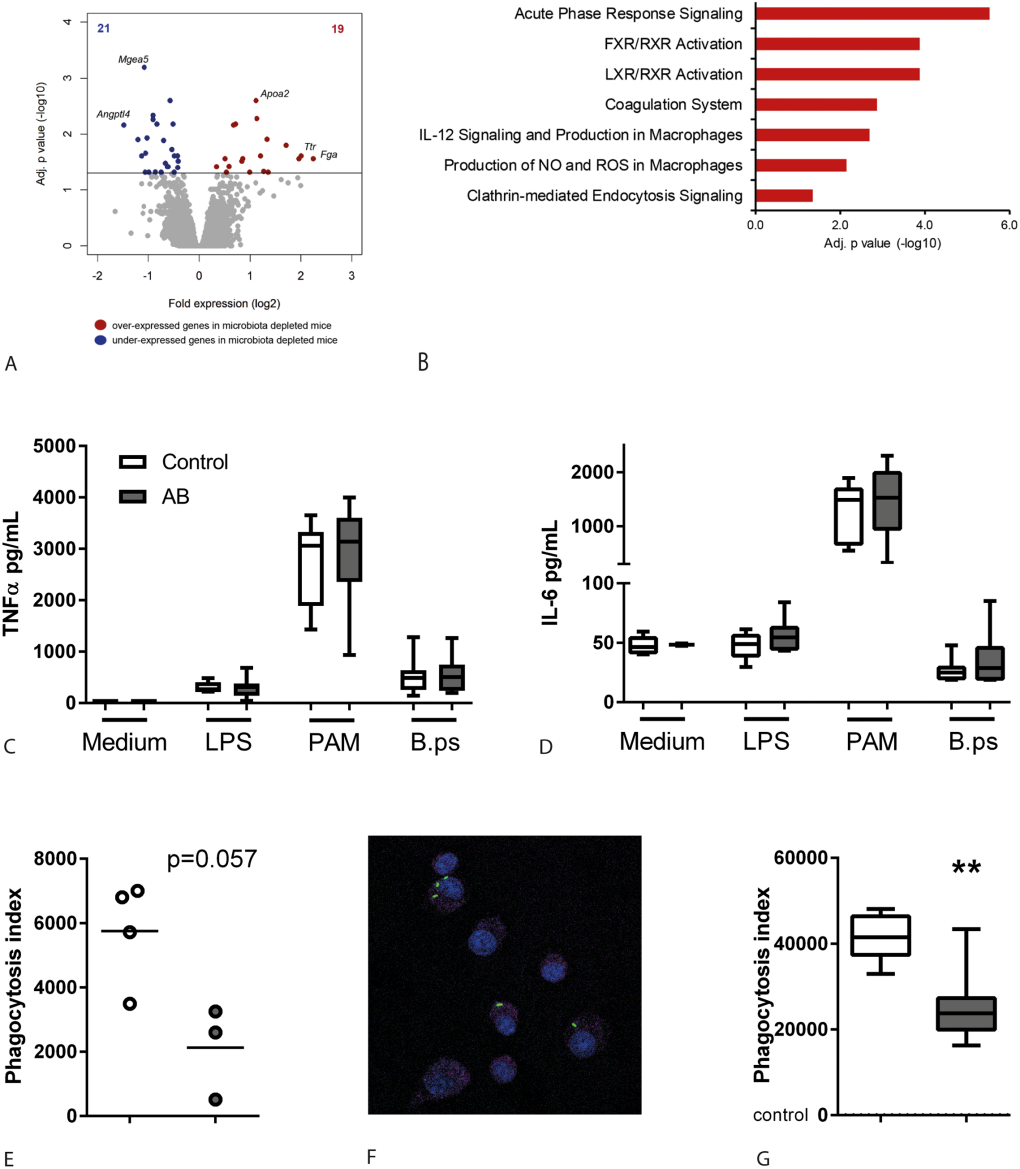
Impaired phagocytosis of *B*. *pseudomallei* by alveolar macrophages derived from gut microbiota disrupted mice. Naïve antibiotic treated- and control mice were sacrificed after the two-day antibiotic washout period and lungs or alveolar macrophages were harvested. (A) Volcano plot depicting significant (multiple comparison adjusted p<0.05) differentially expressed genes in lungs from naïve control and antibiotic pre-treated mice (microbiota disrupted). Red indicates increased expression; blue indicates decreased expression. (B) Significantly enriched canonical signalling pathways in antibiotic treated mice, represented as a bar plot (Ingenuity pathway analysis). (C-D) Alveolar macrophages from naïve control- and antibiotic treated mice were stimulated for 20 hours with medium, LPS 10 ng/mL, PAM3CSK4 1 ug/mL or heat-killed *B*. *pseudomallei* 4x10^6^ CFU/mL and TNFα and IL-6 were measured in supernatant. (E) Alveolar macrophages from naïve control- and antibiotic treated mice (each well pooled from two mice) were incubated with 2,5x107 CFU/mL FITC-labeled heat killed *B*. *pseudomallei* and their phagocytic index determined via flowcytometry as described in the Methods. (F) Control and antibiotic microbiota disrupted mice were given 5x10^6^ heat killed FITC-labeled *B*. *pseudomallei* intranasally and sacrificed after three hours. Cytospins of BALF were stained with PerCP Cy5.5-CD45 and DAPI to assess whether bacteria were located intracellularly (F, representative image). Alveolar macrophages in BALF were analysed by flowcytometry and phagocytic indexes were calculated (G). Data are presented as box- and whisker plots showing the smallest observation, lower quartile, median, upper quartile and largest observation. White bars represent control mice, grey bars antibiotic treated mice. N = 8 mice per group. ** p<0.01 control versus antibiotic treated.

To obtain insight into the mechanism by which the gut microbiota exerts its effects during pneumonia-induced melioidosis, we investigated the effect of antibiotic microbiota disruption on lung transcriptomes. Comparing lung transcriptomes of uninfected intestinal microbiota disrupted mice to control mice revealed 40 significantly altered genes (Fig 5A, 21 genes under-expressed and 19 genes over-expressed in antibiotic pre-treated mice). Ingenuity pathway analysis revealed that genes with elevated expression in antibiotic treated mice significantly enriched several cellular biological pathways, including acute phase response signaling, coagulation system and, notably, IL-12 signaling and production in macrophages as well as production of nitric oxide (NO) and reactive oxygen species (ROS) in macrophages (Fig 5B). Of note, these gene expression differences were not biased by altered neutrophil infiltration (S3 Fig). However, since the analysis was performed on whole lung tissue, it is possible that the genes in these pathways were upregulated in other cell types than macrophages. Altogether, these data suggest that disruption of the intestinal microbiota by antibiotic treatment may impact on lung homeostasis, with macrophages more likely influenced.

As alveolar macrophages are crucial in the first line of defense during pneumonia and are important in the innate immune response in melioidosis [24], we further studied the influence of gut microbiota disruption on the function of alveolar macrophages. Responsiveness of alveolar macrophages derived from gut microbiota-disrupted mice towards PAM3CSK4, LPS or heat-killed *B*. *pseudomallei* was not different from controls in terms of proinflammatory cytokine production (Fig 5C and 5D). In line, no differences in metabolic profiles of alveolar macrophages derived from naïve control and antibiotic pre-treated mice were observed; for this we used extracellular flux technology, which enables assessment of mitochondrial function in live cells, simultaneously measuring oxygen consumption and glycolysis (S4 Fig). In a last set of experiments, we investigated the influence of gut microbiota disruption on the capacity of alveolar macrophages to phagocytose *B*. *pseudomallei*, since an effect of the gut microbiota hereon has been described previously in a setting of *S*. *pneumoniae*, *S*. *aureus* and *K*. *pneumoniae* infection [10, 12, 16]. Cells from BALF were plated and adhering cells were incubated with heat-killed, FITC-labeled *B*. *pseudomallei*, after which the phagocytosis index was determined by flow cytometry (Fig 5E). To confirm this finding, we inoculated mice with heat-killed, FITC-labeled *B*. *pseudomallei* and performed broncho-alveolar lavage three hours later, followed by flowcytometry. Again, we found that alveolar macrophages derived from antibiotic pre-treated mice had a diminished capacity to phagocytose *B*. *pseudomallei* when compared with controls (Fig 5F and 5G). These data suggest that an unperturbed gut microbiota enhances the capacity of alveolar macrophages to phagocytose *B*. *pseudomallei* in vivo. The observed effect was compartment specific; in contrast to alveolar macrophages, *ex vivo* phagocytosis capacity of blood neutrophils, peritoneal macrophages and bone-marrow derived macrophages derived from gut microbiota-disrupted mice was equal compared with controls, as well as cytokine production (S5 Fig). Of note, we did not observe any differences in pulmonary microbiota composition between control- and antibiotic treated mice (S6 Fig).

## Discussion

To the best of our knowledge, this study is the first to investigate the role of the intestinal microbiota during melioidosis. Our data suggest a bidirectional interplay between intestinal microbiota and innate host defenses against *B*. *pseudomallei*. Firstly, we observed significant changes in fecal microbiota composition during melioidosis, independent of antibiotic treatment. A strikingly similar pattern of increased Proteobacteria, decreased Actinobacteria and increased diversity was observed in all mice. Secondly, a well-balanced gut microbiota appears to have a protective effect during melioidosis, especially when *B*. *pseudomallei* has its first encounter with alveolar macrophages in the lung. Antibiotic disruption of the intestinal microbiota affects the capacity of these cells to internalize the pathogen, which was associated with increased bacterial proliferation and dissemination after 24 hours. In this model, the subsequent effects of a disturbed gut microbiota on distant organ injury and survival were limited.

As far as we know, significant changes in the intestinal microbiota within 72 hours of systemic bacterial infection have never been demonstrated before in any model of sepsis. Human studies describing microbiota perturbation during sepsis are confounded by the universal use of antibiotics [25, 26]. We here demonstrate that the systemic inflammatory response itself can lead to marked alterations in the gut microbiota. Our findings are in line with a recent report on intestinal dysbiosis, caused by influenza infection [27]. Another study associated pulmonary *Mycobacterium tuberculosis* infection in mice with loss of intestinal microbiota diversity after six days, with a subsequent recovery during the following weeks [28]. As virtually no *M*. *tuberculosis* was detected in feces, it was suggested that these changes in intestinal microbiota were due to alterations in the adaptive immune system, which in the tuberculosis model becomes effective at controlling the infection around the same time [28]. We therefore expected to find lower microbial diversity three days after infection with *B*. *pseudomallei*, but observed the opposite. A possible explanation could be the elimination of several “big players” of the gut microbiota by the host immune system during severe systemic bacterial infection, giving way to other bacteria to proliferate.

The amount of data that demonstrates a beneficial effect of the intestinal microbiota on the systemic innate immune system in infection is rapidly expanding. Previous studies described a protective effect of the intestinal microbiota during *E*. *coli* and *L*. *monocytogenes* sepsis via stimulation of granulopoiesis in the bone marrow [14, 15]. Crosstalk between microbiota and bone marrow has been suggested to happen via interleukin-17, -22 and granulocyte colony-stimulating factor (G-CSF) [14, 15]. In addition, neutrophil function is affected in both germ free and antibiotic pre-treated mice, resulting in decreased killing of *S*. *pneumoniae* and *S*. *aureus* [10]. In contrast, we did not find any indications for a central role for neutrophils in the antibacterial effect of the microbiota that we observed during melioidosis. The so-called microbiota-bone marrow axis could be more important in younger mice, as were used in above mentioned studies. Also, many studies use germ free mice, which could display more pronounced phenotypes than antibiotic treated mice. Our findings are in line with earlier reports that suggest a positive effect of healthy intestinal microbiota on alveolar macrophages [11, 12, 16, 29]. As alveolar macrophages constantly adapt to their environment, one can imagine them being affected by the level of circulating compounds derived from the intestinal microbiota (e.g. cell wall components or metabolites). Microbial disturbances may induce an altered phenotype of these cells, leading to decreased phagocytosis of *B*. *pseudomallei*, which in turn may lead to decreased intracellular killing. This is in line with our previous findings in a mouse model of pneumococcal pneumonia, in which we found that phagocytic capacities of alveolar macrophages are affected by antibiotic gut microbiota disruption [16]. We found changes in a cholesterol synthesis pathway in the transcriptome of these alveolar macrophages, which could be important as cholesterol-rich membrane rafts are involved in phagocytosis [30].

This study has a number of limitations. The antibiotics were chosen based on similar experiments in the literature [10, 12, 16]; however, other antibiotic regimens may have different effects. Also, mice from different suppliers could have a different intestinal microbiota and as a result elicit different immune responses, as was recently demonstrated in a mouse model for malaria [31]. In addition, we cannot exclude a direct effect of antibiotics on the host response; however, our data are in line with previous reports on the effect of the gut microbiota on the innate immune response during infection [10, 12, 16]. Alterations in the respiratory tract microbiota could be another contributing factor to the observed phenotype [32]. We did however not find any differences in pulmonary microbiota between control- and antibiotic treated mice, making it less likely that this is of influence. Lastly, the situation in actual melioidosis patients is very different from this experimental murine setting; comorbidities, medications and interindividual differences all may influence a possible interplay between microbiota and innate immune system.

In summary, we observed increased bacterial dissemination in mice with a disrupted gut microbiota during pneumonia-derived *B*. *pseudomallei* sepsis, indicating that the intestinal microbiota improves host defense against melioidosis. Alveolar macrophages from microbiota-disrupted mice showed a diminished capacity to phagocytose *B*. *pseudomallei*. It will be very interesting to study if disruption of the microbiota by antibiotics affects susceptibility to melioidosis. There is evidence for the incidence of severe sepsis being higher after events known to be associated with disturbance of the intestinal microbiota, such as hospitalization for *Clostridium difficile* infection [33]. Hopefully, further research into the interplay between intestinal microbiota and melioidosis will tell us whether and how this knowledge could be used to the advantage of patients.

## Methods

A detailed description of methods is available in the online supplement.

### Mice

Specific pathogen-free C57BL/6 mice were purchased from Charles River (Maastricht, The Netherlands). In selected experiments, antibiotic treatment was started at six weeks of age (see below); infection was induced in all experiments at nine weeks of age. The animals were housed in IVC cages in rooms with a controlled temperature and light cyclus. They were acclimatized for one week prior to usage, and received standard rodent chow and water ad libitum.

### Ethics statement

The Institutional Animal Care and Use Committee of the Academic Medical Center approved all experiments (permit number DIX21, sub-protocols 21BB, 21CJ and 21DJ) and ethical approval was obtained to use *B*. *pseudomallei* strain 1026b for animal experiments (08–150; see Supplemental Methods). *B*. *pseudomallei* strain 1026b strain was received by our lab in 2004 as a kind gift from the Donald E. Woods lab, University of Calgary, Alberta, Canada. Samples were anonymized if applicable. Experiments were carried out in accordance with the Dutch Experiments on Animals Act.

### Induction of melioidosis

Experimental melioidosis was induced by intranasal inoculation with 150 or 500 colony forming units (CFU) of *B*. *pseudomallei* strain 1026b as described [17, 18]. At 24 or 72 hours post-infection, mice were euthanized and sacrificed by bleeding from the heart, after which organs were harvested. For survival studies, mice were observed for 14 days.

### Microbiota analysis

Fresh stool pellets were obtained and stored at -80°C. DNA isolation followed by IS-pro bacterial profiling was performed as described before (IS-diagnostics, Amsterdam, The Netherlands) [19, 20]. In short, the length of the 16S-23S rDNA interspace (IS) region is used to classify bacteria by PCR, combined with phylum-specific fluorescent labelling of PCR primers. IS fragment analysis was performed on an ABI Prism 3500 Genetic Analyzer (Applied Biosystems). Data were analysed with IS-pro proprietary software (IS-diagnostics, Amsterdam, The Netherlands).

### Antibiotic treatment

Mice received broad-spectrum antibiotics (ampicillin 1 g/L; neomycin 1 g/L, both from Sigma, Zwijndrecht, The Netherlands; metronidazole 1 g/L, Sanofi-Aventis, Gouda, The Netherlands and vancomycin 0.5 g/L, Xellia pharmaceuticals, Copenhagen, Denmark) in drinking water for 19 days [10, 16]. This cocktail disrupts the intestinal microbiota and significantly lowers microbial diversity [16]. In selected experiments, only ampicillin and metronidazole were used. After a washout period of two days with normal drinking water, mice were inoculated with *B*. *pseudomallei* or sacrificed naïve. Weights of control- and antibiotic treated mice were equal at the moment of inoculation.

### (In vivo) Phagocytosis and stimulation experiments

Alveolar macrophages, peritoneal macrophages, blood and bone marrow derived macrophages were obtained and incubated as described previously [16, 17, 34, 35]. Briefly, cells were seeded, washed and stimulated overnight with LPS or heat-killed *B*. *pseudomallei*. For in vivo phagocytosis, mice were inoculated intranasally with 5x10^6^ CFU heat-killed, FITC (fluoresceine isothiocyanate)-labeled *B*. *pseudomallei*. After three hours, mice were anesthetized and broncho-alveolar lavage (BAL) was performed. FITC-positivity of alveolar macrophages was determined by FACS analysis. Details are provided in the online supplement.

### Whole lung RNA profiling

RNA was isolated from lung homogenates using the RNeasy mini kit (Qiagen, Venlo, The Netherlands). Biotinylated cRNA was hybridized onto the Illumina MouseRef-8v2 Expression BeadChip and an Illumina iScan array scanner (Eindhoven, The Netherlands) was used to scan samples [16, 36]. Detailed methods are available in the supplemental material.

### Statistical analysis

Differences between groups were analyzed by Mann-Whitney U test. Differences in microbiota diversity over time were analysed by paired t-test. For survival, Kaplan-Meier analysis followed by log-rank test was performed and the clinical scores by matched two-way ANOVA. Analyses were performed using GraphPad Prism 5. Values of P<0.05 were considered statistically significant.

## Supporting information

S1 Fig Antibiotic pre-treatment does not affect *B*. *pseudomallei* loads in broncho-alveolar lavage fluid during experimental melioidosis. Control and antibiotic pre-treated mice were inoculated intranasally with 150 CFU (A) or 500 CFU (B) *B*. *pseudomallei* and sacrificed at the indicated time points. Bacterial loads in broncho-alveolar lavage fluid (BALF) are depicted as scatter dot plots with a line at the median. White dots represent control mice, grey dots antibiotic treated mice. N = 6–8 mice per group.
(TIF)

S2 Fig Ampicillin and metronidazole pre-treatment is associated with decreased pulmonary clearance of *B*. *pseudomallei* during experimental melioidosis. Mice were pre-treated with metronidazole and ampicillin in drinking water, in exactly the same experimental setup as described in the Methods section. Control and antibiotic pre-treated mice were inoculated intranasally with 500 CFU *B*. *pseudomallei* and sacrificed 24 hours after infection. Bacterial loads in blood, liver and spleen homogenate (A) and lung homogenate (B) are depicted as scatter dot plots with a line at the median. White dots represent control mice, grey dots antibiotic treated mice. N = 8 mice per group. *p<0.05 control versus antibiotic treated.
(TIF)

S3 Fig Cellular composition of broncho-alveolar lavage fluid from naïve control and antibiotic pre-treated mice. Bar chart depicting the median percentage of alveolar macrophages (AMs) and neutrophils in broncho-alveolar lavage fluid from naïve control and antibiotic pre-treated mice.
(TIF)

S4 Fig Cellular metabolism of alveolar macrophages from control and antibiotic pre-treated mice. (A) Extracellular acidification rate (ECAR) and (B) oxygen consumption rate (OCR) of alveolar macrophages from control and antibiotic treated mice, as a measure for glycolytic function and mitochondrial respiration function, respectively. Arrows indicate the sequential adding of oligomycin (O, 1,5 μM), FCCP (F, 1,5 μM) and antimycin A + rotenone (A/R, 2,5 μM/ 1,25 μM). Data are normalized to DNA content. White dots represent control mice, grey antibiotic treated mice. Data are presented as median ± interquartile range (n = 6–7 per group).
(TIF)

S5 Fig Bone marrow derived macrophages, peritoneal macrophages and blood from control and antibiotic pre-treated mice do not differ in terms of cellular responsiveness or phagocytosis capacity. Naïve antibiotic treated- and control mice were sacrificed after the two-day antibiotic washout period and bone marrow, blood and peritoneal macrophages were harvested. Bone marrow derived macrophages (BMDM), peritoneal macrophages and blood were stimulated with LPS 100 ng/mL or 3x10^7^ CFU/mL heat-killed *B*. *pseudomallei* for 14 hours (A-B, D-E, G-H). Alternatively, whole blood or macrophages were incubated with 2,5x10^7^ CFU/mL FITC-labeled heat killed *B*. *pseudomallei* to investigate their phagocytic capacities (C, F, I). Internalization of bacteria was assessed by flowcytometry as described in the Methods section. TNF-α and IL-6 production by BMDMs (A-B), peritoneal macrophages (D-E) and whole blood (G-H) upon stimulation with LPS or heat-killed *B*. *pseudomallei* did not differ between groups. The phagocytic capacity of BMDMs (C), peritoneal macrophages (F) and whole blood derived neutrophils was also similar (I). Data are presented as box- and whisker plots showing the smallest observation, lower quartile, median, upper quartile and largest observation. White bars represent control mice, grey bars antibiotic treated mice. N = 4–8 mice per group. All are representative of two experiments.
(TIF)

S6 Fig Composition of pulmonary microbiota in naïve control and antibiotic pre-treated mice. To investigate the pulmonary microbiota, whole lungs were harvested from naïve control and antibiotic (ampicillin, vancomycin, metronidazole and neomycin) pre-treated mice on day 19. Total bacterial 16S rDNA was isolated and sequenced. (A) Lung microbiota composition at the Class level (second highest bacterial taxonomic rank). (B) Lung microbiota composition at the Genus level (lowest detectable bacterial taxonomic rank). Data are presented as box-and-whisker plots depicting median, interquartile range and range of the percentage of total 16S rDNA reads in each sample (n = 6 mice/group). White, control mice; grey, antibiotic pre-treated mice. Only Classes and Genera that had a median prevalence of 5% or more amongst all samples are depicted. No statistically significant differences were detected.
(TIF)

S1 Methods Supplemental methods. (DOCX)

## References

[1] Wiersinga WJ, Currie BJ, Peacock SJ. Melioidosis. The New England journal of medicine. 2012;367(11):1035–44. Epub 2012/09/14. 10.1056/NEJMra1204699 22970946

[2] Currie BJ. Melioidosis: evolving concepts in epidemiology, pathogenesis, and treatment. Semin Respir Crit Care Med. 2015;36(1):111–25. 10.1055/s-0034-1398389 25643275

[3] Meumann EM, Cheng AC, Ward L, Currie BJ. Clinical features and epidemiology of melioidosis pneumonia: results from a 21-year study and review of the literature. Clinical infectious diseases: an official publication of the Infectious Diseases Society of America. 2012;54(3):362–9. 22057702 10.1093/cid/cir808 PMC3258273

[4] Birnie E, Wiersinga WJ, Limmathurotsakul D, Grobusch MP. Melioidosis in Africa: should we be looking more closely? Future Microbiol. 2015;10(2):273–81. 10.2217/fmb.14.113 25689538

[5] Limmathurotsakul D, Golding N, Dance DAB, Messina JP, Pigott DM, Moyes CL, et al Predicted global distribution of Burkholderia pseudomallei and burden of melioidosis. Nature Microbiology. 2016;1:15008. 10.1038/nmicrobiol.2015.8 27571754

[6] Schweizer HP. Mechanisms of antibiotic resistance in Burkholderia pseudomallei: implications for treatment of melioidosis. Future Microbiol. 2012;7(12):1389–99. 10.2217/fmb.12.116 23231488 PMC3568953

[7] Blaser M, Bork P, Fraser C, Knight R, Wang J. The microbiome explored: recent insights and future challenges. Nat Rev Microbiol. 2013;11(3):213–7. 10.1038/nrmicro2973 23377500

[8] Pamer EG. Resurrecting the intestinal microbiota to combat antibiotic-resistant pathogens. Science. 2016;352(6285):535–8. 10.1126/science.aad9382 27126035 PMC4984266

[9] Schuijt TJ, van der Poll T, de Vos WM, Wiersinga WJ. The intestinal microbiota and host immune interactions in the critically ill. Trends in microbiology. 2013;21(5):221–9. Epub 2013/03/05. 10.1016/j.tim.2013.02.001 23454077

[10] Clarke TB, Davis KM, Lysenko ES, Zhou AY, Yu Y, Weiser JN. Recognition of peptidoglycan from the microbiota by Nod1 enhances systemic innate immunity. Nature medicine. 2010;16(2):228–31. Epub 2010/01/19. 10.1038/nm.2087 20081863 PMC4497535

[11] Bernard H, Desseyn JL, Bartke N, Kleinjans L, Stahl B, Belzer C, et al Dietary pectin-derived acidic oligosaccharides improve the pulmonary bacterial clearance of Pseudomonas aeruginosa lung infection in mice by modulating intestinal microbiota and immunity. The Journal of infectious diseases. 2015;211(1):156–65. Epub 2014/08/21. 10.1093/infdis/jiu391 25139019

[12] Clarke TB. Early innate immunity to bacterial infection in the lung is regulated systemically by the commensal microbiota via nod-like receptor ligands. Infection and immunity. 2014;82(11):4596–606. Epub 2014/08/20. 10.1128/IAI.02212-14 25135683 PMC4249320

[13] Gauguet S, D'Ortona S, Ahnger-Pier K, Duan B, Surana NK, Lu R, et al Intestinal Microbiota of Mice Influences Resistance to Staphylococcus aureus Pneumonia. Infection and immunity. 2015;83(10):4003–14. Epub 2015/07/29. 10.1128/IAI.00037-15 26216419 PMC4567647

[14] Deshmukh HS, Liu Y, Menkiti OR, Mei J, Dai N, O'Leary CE, et al The microbiota regulates neutrophil homeostasis and host resistance to Escherichia coli K1 sepsis in neonatal mice. Nature medicine. 2014;20(5):524–30. Epub 2014/04/22. 10.1038/nm.3542 24747744 PMC4016187

[15] Khosravi A, Yanez A, Price JG, Chow A, Merad M, Goodridge HS, et al Gut microbiota promote hematopoiesis to control bacterial infection. Cell host & microbe. 2014;15(3):374–81. Epub 2014/03/19. 24629343 10.1016/j.chom.2014.02.006 PMC4144825

[16] Schuijt TJ, Lankelma JM, Scicluna BP, de Sousa EMF, Roelofs JJ, de Boer JD, et al The gut microbiota plays a protective role in the host defence against pneumococcal pneumonia. Gut. 2016;65(4):575–83. 10.1136/gutjnl-2015-309728 26511795 PMC4819612

[17] Weehuizen TA, Hommes TJ, Lankelma JM, de Jong HK, Roelofs JJ, de Vos AF, et al Triggering Receptor Expressed on Myeloid Cells (TREM)-2 Impairs Host Defense in Experimental Melioidosis. PLoS neglected tropical diseases. 2016;10(6):e0004747 10.1371/journal.pntd.0004747 27253382 PMC4890812

[18] Wiersinga WJ, Wieland CW, Dessing MC, Chantratita N, Cheng AC, Limmathurotsakul D, et al Toll-like receptor 2 impairs host defense in gram-negative sepsis caused by Burkholderia pseudomallei (Melioidosis). PLoS Med. 2007;4(7):e248 10.1371/journal.pmed.0040248 17676990 PMC1950213

[19] Budding AE, Grasman ME, Lin F, Bogaards JA, Soeltan-Kaersenhout DJ, Vandenbroucke-Grauls CM, et al IS-pro: high-throughput molecular fingerprinting of the intestinal microbiota. FASEB journal: official publication of the Federation of American Societies for Experimental Biology. 2010;24(11):4556–64. Epub 2010/07/21. 20643909 10.1096/fj.10-156190

[20] de Meij TG, Budding AE, de Groot EF, Jansen FM, Frank Kneepkens CM, Benninga MA, et al Composition and stability of intestinal microbiota of healthy children within a Dutch population. FASEB journal: official publication of the Federation of American Societies for Experimental Biology. 2016;30(4):1512–22. 26655704 10.1096/fj.15-278622

[21] Zhang D, Chen G, Manwani D, Mortha A, Xu C, Faith JJ, et al Neutrophil ageing is regulated by the microbiome. Nature. 2015;525(7570):528–32. Epub 2015/09/17. 10.1038/nature15367 26374999 PMC4712631

[22] Easton A, Haque A, Chu K, Lukaszewski R, Bancroft GJ. A critical role for neutrophils in resistance to experimental infection with Burkholderia pseudomallei. The Journal of infectious diseases. 2007;195(1):99–107. 10.1086/509810 17152013

[23] Wiersinga WJ, Veer C, Wieland CW, Gibot S, Hooibrink B, Day NP, et al Expression profile and function of triggering receptor expressed on myeloid cells-1 during melioidosis. The Journal of infectious diseases. 2007;196(11):1707–16. 10.1086/522141 18008257

[24] Wiersinga WJ, van der Poll T, White NJ, Day NP, Peacock SJ. Melioidosis: insights into the pathogenicity of Burkholderia pseudomallei. Nat Rev Microbiol. 2006;4(4):272–82. 10.1038/nrmicro1385 16541135

[25] Ojima M, Motooka D, Shimizu K, Gotoh K, Shintani A, Yoshiya K, et al Metagenomic Analysis Reveals Dynamic Changes of Whole Gut Microbiota in the Acute Phase of Intensive Care Unit Patients. Digestive diseases and sciences. 2016;61(6):1628–34. 10.1007/s10620-015-4011-3 26715502 PMC4875048

[26] Zaborin A, Smith D, Garfield K, Quensen J, Shakhsheer B, Kade M, et al Membership and behavior of ultra-low-diversity pathogen communities present in the gut of humans during prolonged critical illness. MBio. 2014;5(5):e01361–14. 10.1128/mBio.01361-14 25249279 PMC4173762

[27] Deriu E, Boxx GM, He X, Pan C, Benavidez SD, Cen L, et al Influenza Virus Affects Intestinal Microbiota and Secondary Salmonella Infection in the Gut through Type I Interferons. PLoS Pathog. 2016;12(5):e1005572 10.1371/journal.ppat.1005572 27149619 PMC4858270

[28] Winglee K, Eloe-Fadrosh E, Gupta S, Guo H, Fraser C, Bishai W. Aerosol Mycobacterium tuberculosis infection causes rapid loss of diversity in gut microbiota. PloS one. 2014;9(5):e97048 10.1371/journal.pone.0097048 24819223 PMC4018338

[29] Kim YG, Udayanga KG, Totsuka N, Weinberg JB, Nunez G, Shibuya A. Gut dysbiosis promotes M2 macrophage polarization and allergic airway inflammation via fungi-induced PGE(2). Cell host & microbe. 2014;15(1):95–102. Epub 2014/01/21. 24439901 10.1016/j.chom.2013.12.010 PMC3957200

[30] Kannan S, Audet A, Huang H, Chen LJ, Wu M. Cholesterol-rich membrane rafts and Lyn are involved in phagocytosis during Pseudomonas aeruginosa infection. J Immunol. 2008;180(4):2396–408. 18250449 10.4049/jimmunol.180.4.2396

[31] Villarino NF, LeCleir GR, Denny JE, Dearth SP, Harding CL, Sloan SS, et al Composition of the gut microbiota modulates the severity of malaria. Proceedings of the National Academy of Sciences of the United States of America. 2016;113(8):2235–40. 10.1073/pnas.1504887113 26858424 PMC4776451

[32] Dickson RP, Huffnagle GB. The Lung Microbiome: New Principles for Respiratory Bacteriology in Health and Disease. PLoS Pathog. 2015;11(7):e1004923 10.1371/journal.ppat.1004923 26158874 PMC4497592

[33] Prescott HC, Dickson RP, Rogers MA, Langa KM, Iwashyna TJ. Hospitalization Type and Subsequent Severe Sepsis. American journal of respiratory and critical care medicine. 2015;192(5):581–8. Epub 2015/05/29. 10.1164/rccm.201503-0483OC 26016947 PMC4595694

[34] Koh GC, Weehuizen TA, Breitbach K, Krause K, de Jong HK, Kager LM, et al Glyburide reduces bacterial dissemination in a mouse model of melioidosis. PLoS neglected tropical diseases. 2013;7(10):e2500 Epub 2013/10/23. 10.1371/journal.pntd.0002500 24147174 PMC3798430

[35] Wiersinga WJ, Kager LM, Hovius JW, van der Windt GJ, de Vos AF, Meijers JC, et al Urokinase receptor is necessary for bacterial defense against pneumonia-derived septic melioidosis by facilitating phagocytosis. J Immunol. 2010;184(6):3079–86. 10.4049/jimmunol.0901008 20142364

[36] van Lieshout MH, Scicluna BP, Florquin S, van der Poll T. NLRP3 and ASC differentially affect the lung transcriptome during pneumococcal pneumonia. American journal of respiratory cell and molecular biology. 2014;50(4):699–712. 10.1165/rcmb.2013-0015OC 24164497

