# Supplementary figures and images for: The gut microbiota as a modulator of innate immunity during melioidosis
Source: PLoS Negl Trop Dis. 2017 Apr 19;11(4):e0005548. doi: 10.1371/journal.pntd.0005548 (PMC5411098; doi:10.1371/journal.pntd.0005548)

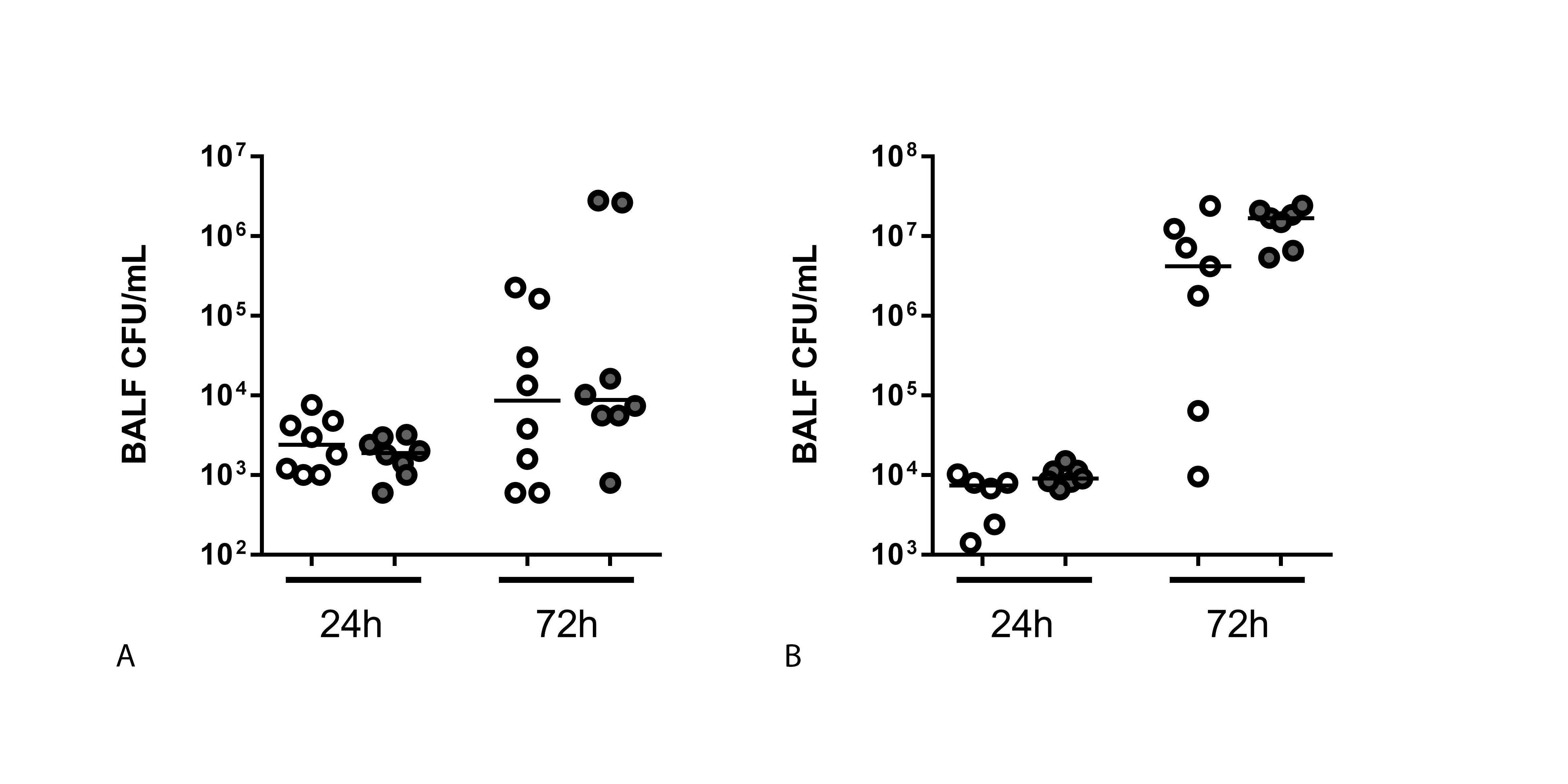

Supplement: S1 Fig — Control and antibiotic pre-treated mice were inoculated intranasally with 150 CFU (A) or 500 CFU (B) B. pseudomallei and sacrificed at the indicated time points. Bacterial loads in broncho-alveolar lavage fluid (BALF) are depicted as scatter dot plots with a line at the median. White dots represent control mice, grey dots antibiotic treated mice. N = 6–8 mice per group. (TIF) [file pntd.0005548.s001.tif]

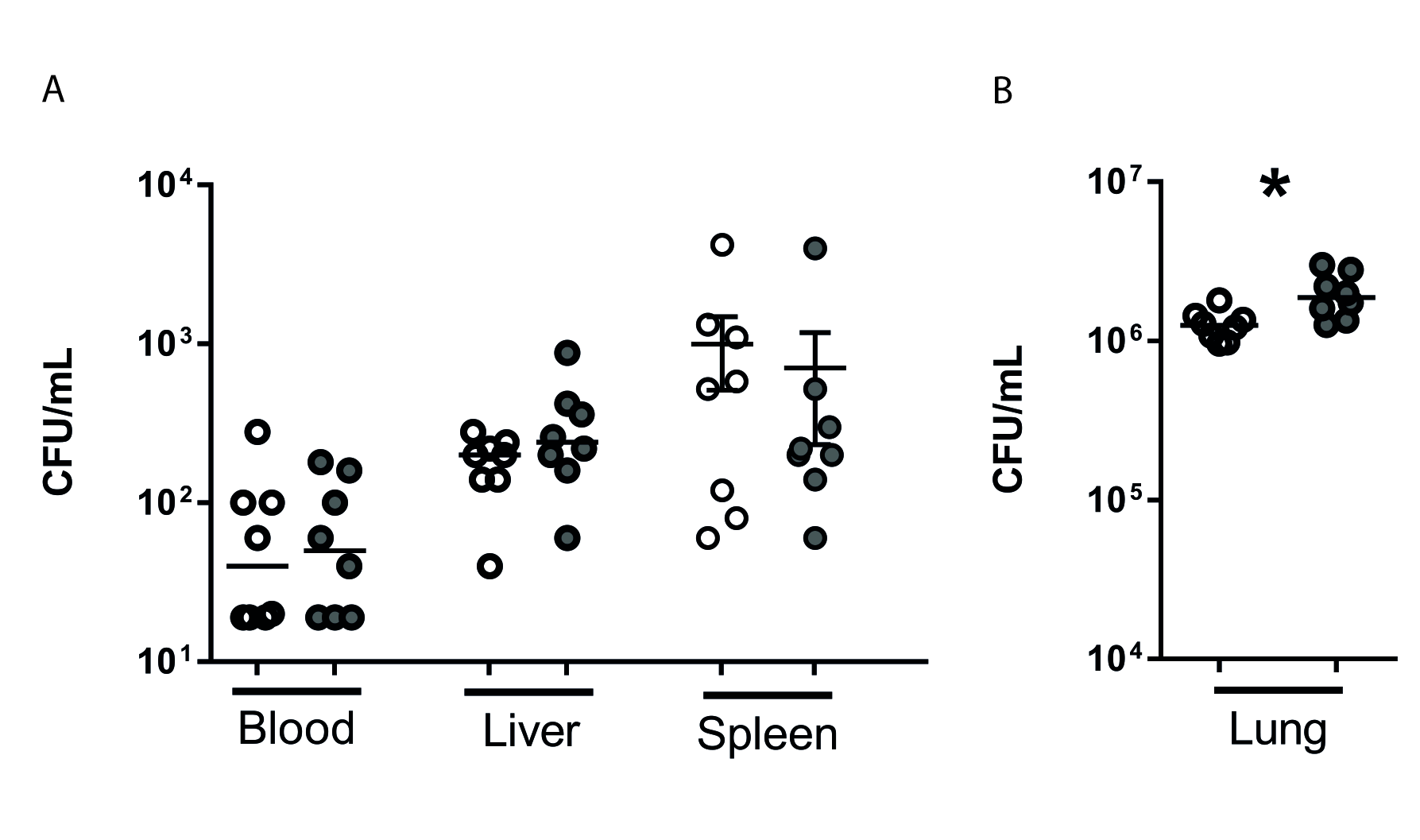

Supplement: S2 Fig — Mice were pre-treated with metronidazole and ampicillin in drinking water, in exactly the same experimental setup as described in the Methods section. Control and antibiotic pre-treated mice were inoculated intranasally with 500 CFU B. pseudomallei and sacrificed 24 hours after infection. Bacterial loads in blood, liver and spleen homogenate (A) and lung homogenate (B) are depicted as scatter dot plots with a line at the median. White dots represent control mice, grey dots antibiotic treated mice. N = 8 mice per group. *p<0.05 control versus antibiotic treated. (TIF) [file pntd.0005548.s002.tif]

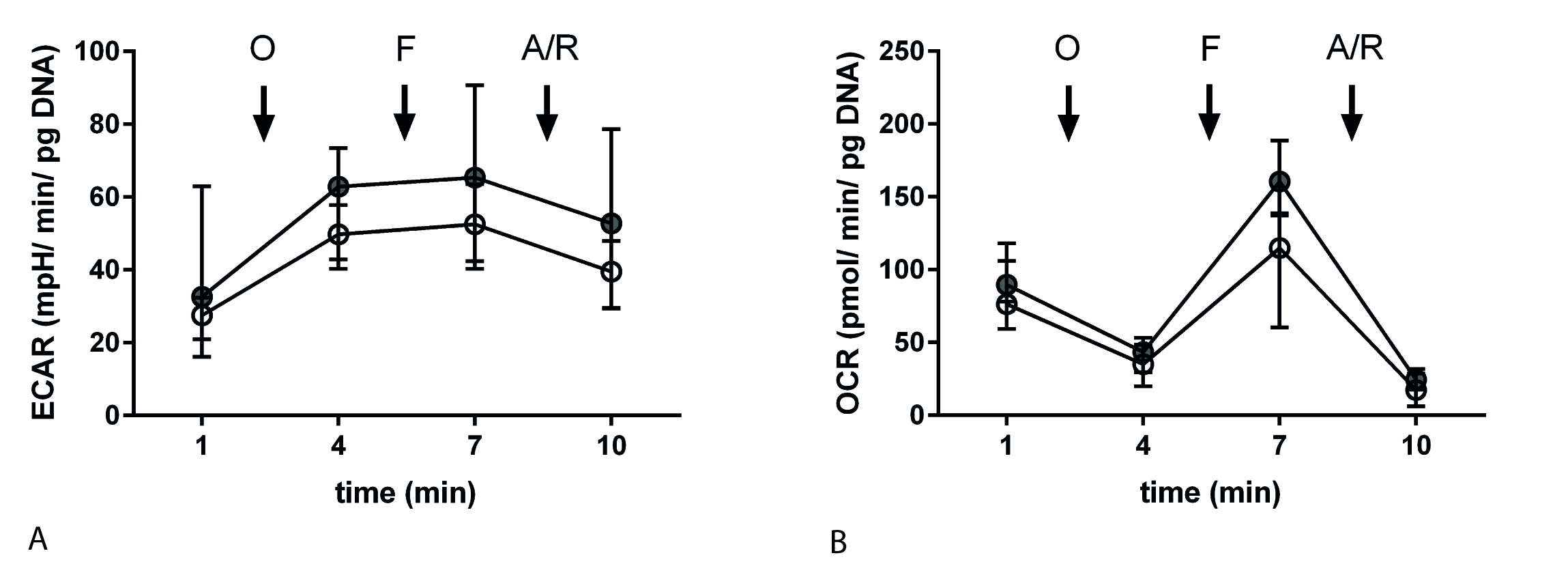

Supplement: S4 Fig — (A) Extracellular acidification rate (ECAR) and (B) oxygen consumption rate (OCR) of alveolar macrophages from control and antibiotic treated mice, as a measure for glycolytic function and mitochondrial respiration function, respectively. Arrows indicate the sequential adding of oligomycin (O, 1,5 μM), FCCP (F, 1,5 μM) and antimycin A + rotenone (A/R, 2,5 μM/ 1,25 μM). Data are normalized to DNA content. White dots represent control mice, grey antibiotic treated mice. Data are presented as median ± interquartile range (n = 6–7 per group). (TIF) [file pntd.0005548.s004.tif]

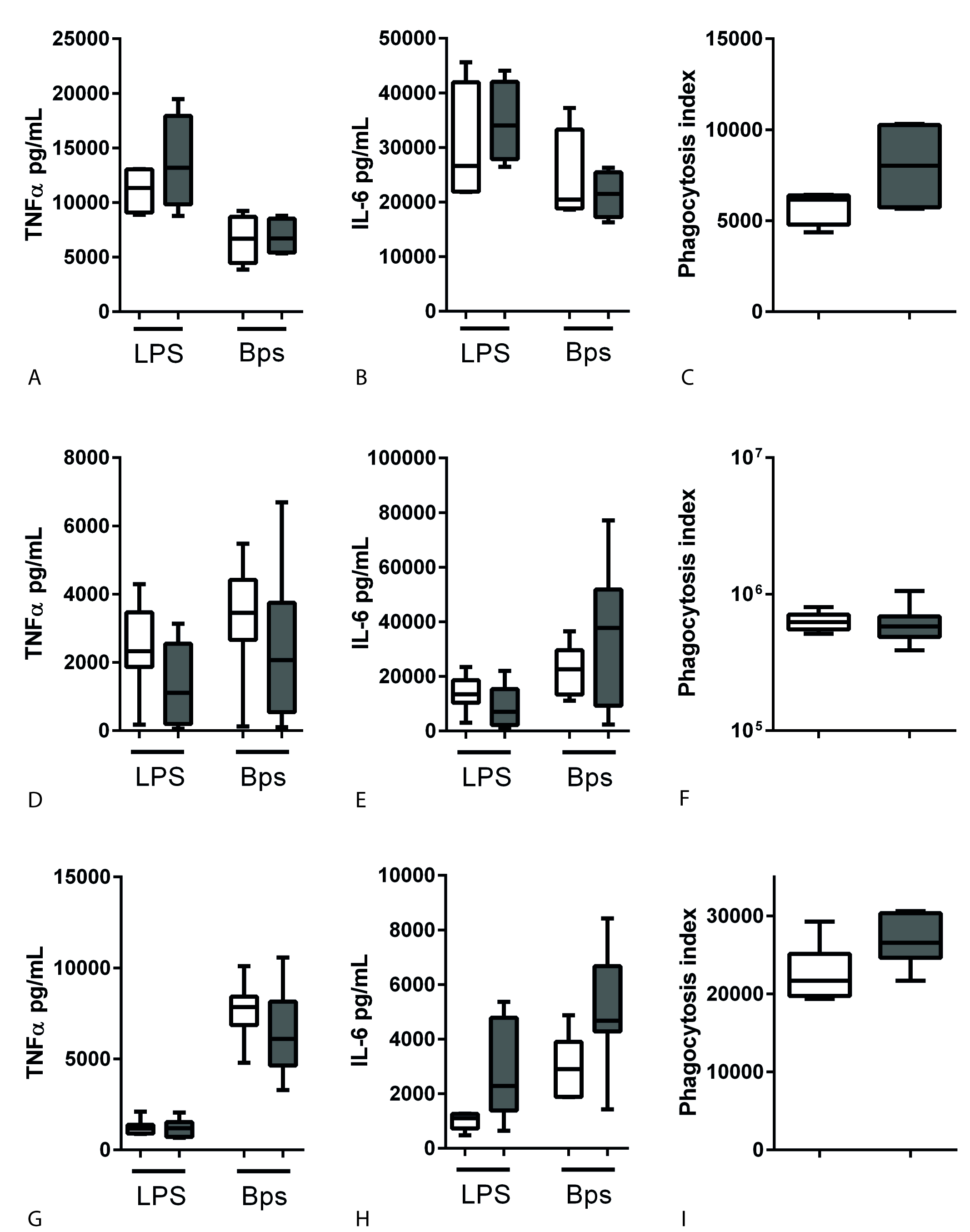

Supplement: S5 Fig — Naïve antibiotic treated- and control mice were sacrificed after the two-day antibiotic washout period and bone marrow, blood and peritoneal macrophages were harvested. Bone marrow derived macrophages (BMDM), peritoneal macrophages and blood were stimulated with LPS 100 ng/mL or 3x10^7^ CFU/mL heat-killed B. pseudomallei for 14 hours (A-B, D-E, G-H). Alternatively, whole blood or macrophages were incubated with 2,5x10^7^ CFU/mL FITC-labeled heat killed B. pseudomallei to investigate their phagocytic capacities (C, F, I). Internalization of bacteria was assessed by flowcytometry as described in the Methods section. TNF-α and IL-6 production by BMDMs (A-B), peritoneal macrophages (D-E) and whole blood (G-H) upon stimulation with LPS or heat-killed B. pseudomallei did not differ between groups. The phagocytic capacity of BMDMs (C), peritoneal macrophages (F) and whole blood derived neutrophils was also similar (I). Data are presented as box- and whisker plots showing the smallest observation, lower quartile, median, upper quartile and largest observation. White bars represent control mice, grey bars antibiotic treated mice. N = 4–8 mice per group. All are representative of two experiments. (TIF) [file pntd.0005548.s005.tif]

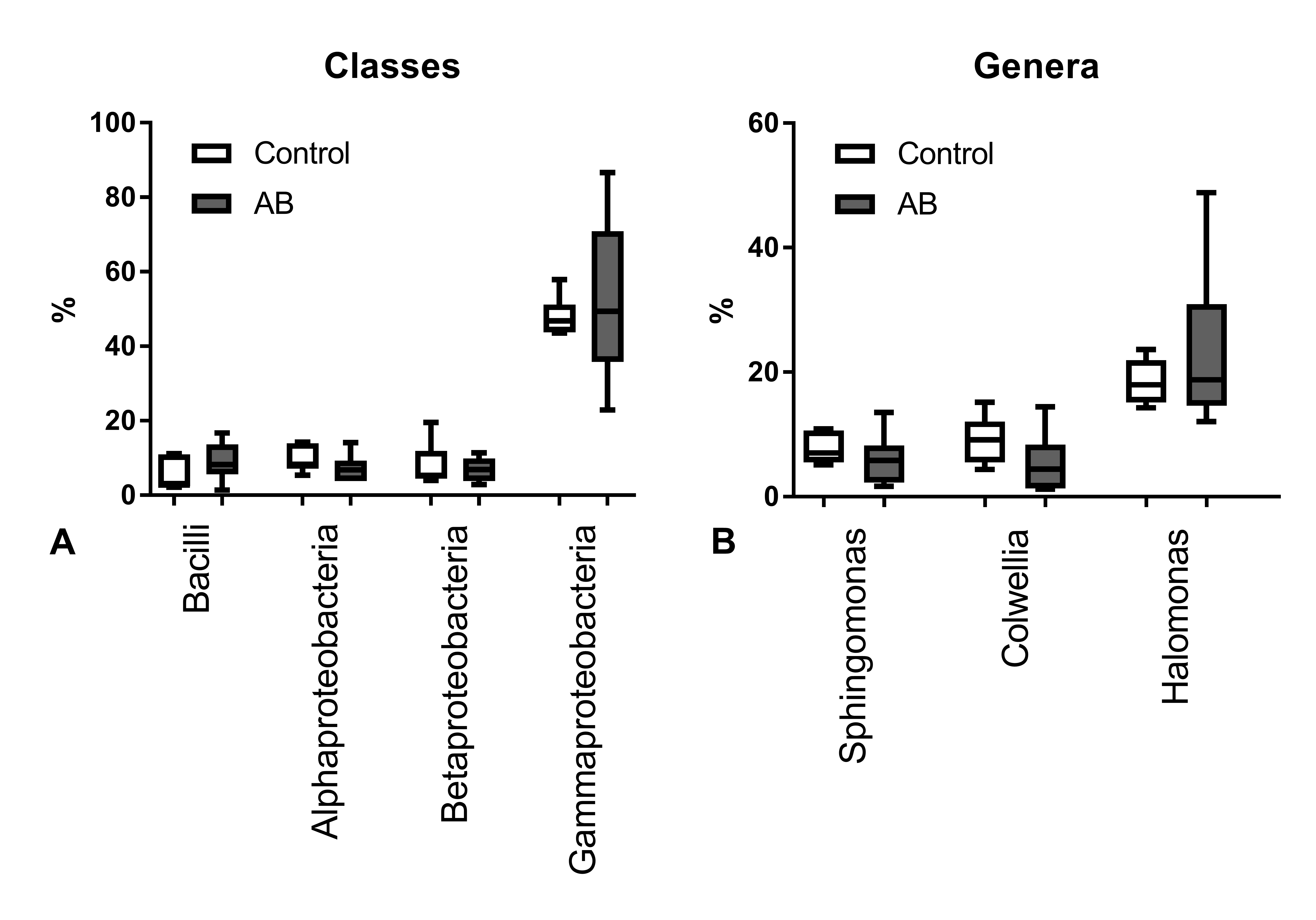

Supplement: S6 Fig — To investigate the pulmonary microbiota, whole lungs were harvested from naïve control and antibiotic (ampicillin, vancomycin, metronidazole and neomycin) pre-treated mice on day 19. Total bacterial 16S rDNA was isolated and sequenced. (A) Lung microbiota composition at the Class level (second highest bacterial taxonomic rank). (B) Lung microbiota composition at the Genus level (lowest detectable bacterial taxonomic rank). Data are presented as box-and-whisker plots depicting median, interquartile range and range of the percentage of total 16S rDNA reads in each sample (n = 6 mice/group). White, control mice; grey, antibiotic pre-treated mice. Only Classes and Genera that had a median prevalence of 5% or more amongst all samples are depicted. No statistically significant differences were detected. (TIF) [file pntd.0005548.s006.tif]
