# Supplementary material for: The gut microbiota as a modulator of innate immunity during melioidosis
Source: PLoS Negl Trop Dis. 2017 Apr 19;11(4):e0005548. doi: 10.1371/journal.pntd.0005548 (PMC5411098; doi:10.1371/journal.pntd.0005548)
Supplement: S1 Methods — (DOCX) [file pntd.0005548.s007.docx]

**S1 Methods**

**The Gut Microbiota as a Modulator of Innate Immunity during Melioidosis**

Jacqueline M. Lankelma, Emma Birnie, Tassili A. F. Weehuizen, Brendon Scicluna, Clara Belzer, Riekelt H. Houtkooper, Joris J. T. H. Roelofs, Alex F. de Vos, Andries E. Budding, W. Joost Wiersinga

**Experimental infection**

*B. pseudomallei* strain 1026b, isolated in 1993 from a blood culture from a septic 29-year old female rice farmer in Sappasithiprasong hospital, was grown from frozen aliquots in 50 mL Luria Broth overnight at 37°C in a shaking 5% CO2 incubator [[1](#_ENREF_1), [2](#_ENREF_2)]. Two mL of this culture was transferred to fresh Luria Broth and grown for about three hours to midlogarithmic phase. Bacteria were diluted in sterile saline to a concentration of 150, 500 or 750 CFU/ 50 uL, as determined by plating serial dilutions on blood agar plates. Pneumonia was induced by intranasal inoculation of 50 uL bacterial suspension after inhalation anesthesia with isoflurane (2-3% in 100% oxygen) as described [[1](#_ENREF_1), [2](#_ENREF_2)].

**Clinical observation score**

Clinical signs were scored as previously described [[3](#_ENREF_3)]: solitude (0 absent, 1 present), posture (0 normal, 1 sphere), fur (0 normal, 1 pilo‐erection), eyes (0 open, 1 closed, 2 dirty), alertness (0 normal, 1 slow, 2 apathic, 3 non‐responsive), pace (0 normal, 1 shaky, 2 collapse), respiration (0 normal, 1 heavy, 2 slow, 3 intermittent) and time to ascent when laid down (0 normal, 1 <5 seconds, 2 >5 seconds, 3 unresponsive); resulting in a maximum score of 16. Dead mice were given the highest clinical score.

**Sample collection and determination of bacterial loads**

Mice were euthanized by intraperitoneal injection of ketamine/ dexmedetomidine 24 or 72 hours after induction of pneumonia (n=6-8 mice per group) or observed for 14 days (n=20 per group). Blood was drawn by cardiac puncture, transferred to heparin Vacutainer tubes (BD Bioscience, Breda, The Netherlands), and immediately cooled. Broncho-alveolar lavage (BAL) was performed using 1 mL phosphate buffered saline (PBS). Next, the abdomen was opened and liver and lungs were harvested. Lungs and liver were homogenised in four volumes of sterile isotonic saline. For bacterial quantification, blood and organ homogenates were serially 10-fold diluted in sterile saline and 50 uL of each dilution was plated onto blood agar plates. Following 24h of incubation at 37°C, CFUs were counted and corrected for dilution. For cytokine measurements, lung homogenates were diluted 1:1 with Greenberger lysis buffer (300 mM NaCl, 30 mM Tris, 2 mM MgCl2, 2 mM CaCl2, 1% (v/v) Triton X-100, pH 7.4) with protease inhibitor mix (Complete protease inhibitor cocktail tablets, Roche, Almere, The Netherlands) and incubated for 30 min on ice, followed by centrifugation at 1750 g for 10 min. Supernatants were stored at -20°C until analysis.

**Histology**

Directly after sacrifice, lungs were fixed in 4% formalin and embedded in paraffin for routine histology. Sections of 4um thickness were stained with haematoxylin and eosin. All slides were scored by an experienced, blinded pathologist. Lungs were graded for bronchitis, edema, interstitial inflammation, necrosis, pleuritis, endothelialitis and percentage of slide surface involved on a scale of 0 to 4, with 0 as “absent” and 4 as “severe”. The total lung inflammation score is expressed as the sum of the scores for each parameter. Granulocyte staining was performed using FITC-labelled rat anti-mouse Ly-6GC mAb (BD Bioscience, Breda, The Netherlands), counterstained with methylgreen. Lung tissue sections were scanned with an Olympus Slide system and Ly-6GC staining was quantified by digital image analysis (ImageJ); the amount of Ly-6GC positivity is expressed as a percentage of the total lung surface.

**Assays**

Tumor necrosis factor (TNF)-α, interferon (IFN)-γ, interleukin (IL)-6 and CCL2 were measured in plasma by cytometric bead array (mouse inflammation kit, BD Biosciences) in accordance with the manufacturers’ recommendations. TNFα, IL-1β, IL-6 and CXCL1 in lung homogenate were determined by enzyme linked immunosorbent assays (ELISA; R&D Systems, Minneapolis, MN). Lactate dehydrogenase (LDH), urea, aspartate aminotranspherase (AST) and alanine aminotranspherase (ALT) were measured using a c702 Roche Diagnostics machine.

**Cellular stimulation, phagocytosis & killing experiments**

Bone marrow was harvested from femurs of naïve control- and antibiotic treated mice and BMDMs were generated as described before [[4](#_ENREF_4)]. Essentially, bone marrow was cultured in presence of 2 ng/mL GM-CSF, Panexin BMM 5% v/v (PAN-Biotech, Aidenbach, Germany) and 50 mM 2-mercaptoethanol for one week. Macrophages were seeded in 96-wells plates, adhered overnight and washed (alveolar macrophages 40.000 cells/well; BMDMs and peritoneal macrophages 50.000 cells/well). Whole blood (100 uL/well) or macrophages were stimulated with LPS 100 ng/mL (Invivogen, San Diego, California) or 2,5x10^7^ heat-killed *B. pseudomallei*/mL for 20 hours. TNF-α and IL-6 in supernatant were measured by ELISA (R&D Systems, Minneapolis, MN). A phagocytosis assay was performed by incubating whole blood, BMDMs, alveolar macrophages or peritoneal macrophages (plated as above) with 2.5x10^7^heat-killed FITC-labeled *B. pseudomallei*/mL at 37°C. After two hours, macrophages were placed on ice, washed with ice cold PBS, mobilized by scraping, quenched with trypan blue and washed. After 30 minutes, blood was washed and incubated with erythrocyte lysis buffer (155 mM NH4Cl, 10 mM KHCO3, and 0.1 mM EDTA, pH 7.4) for 15 min on ice. Samples were analysed on a FACS Calibur or FACS Canto II (BD Bioscience, Breda, The Netherlands). In all experiments, a phagocytosis index of each sample was calculated as mean fluorescence intensity (MFI) of positive cells × percentage (%) positive cells, followed by subtraction of the value of a corresponding sample that was kept on ice.

**In vivo alveolar macrophage phagocytosis**

Mice were inoculated intranasally with 5x10^6^ CFU heat-killed, FITC (fluoresceine isothiocyanate)-labeled *B. pseudomallei*. After three hours, broncho-alveolar lavage was performed with 5 mL sterile PBS with 2 mM EDTA. BALF was centrifuged at 400G for 10 minutes, after which cells were resuspended in 200 uL PBS and counted using a Coulter Counter. After incubation with an Fc blocking antibody (anti mouse CD16/32, eBioscience, 93), cells were stained with the following antibodies: PE Ly-6GC (BD Bioscience, RB6-8C5), PE-Efluor 610 CD45.2 (eBioscience, 30-F11), PE-Cy7 CD11b (BD Bioscience, M1/70), Alexa fluor 647 Siglec F (BD Bioscience, E50-2440), PerCP-Cy5.5 CD11c (BD Bioscience, HL3) and eFluor 780 viability dye (eBioscience). Cells were fixed with 4% paraformaldehyde, washed and analysed using a FACS Canto II. Alveolar macrophages were defined as viable (CD45+, CD11c+, SiglecF+). Cytospins of BALF were fixed with 4% paraformaldehyde, washed twice with PBS and stained with PerCP Cy5.5 CD45 (EBioscience, 30-F11) in 0.1% Triton in PBS. After washing three times, Prolong Gold with DAPI (Thermo Fisher) was added and after drying, samples were analysed using a Leica TCS SP8 X mounted on a Leica DMI6000 inverted microscope.

**FACS analysis**

In short, after erylysis, washing and incubation with an Fc blocking antibody (anti mouse CD16/32, eBioscience, clone 93), bone marrow and blood cells were stained with the following antibodies: PE Ly-6G (BD Bioscience, 1A8), FITC CD45R/B220 (BD Bioscience, RA3-6B2), PE-Efluor 610-CD45.2 (eBioscience, 30-F11), Alexa Fluor 700 CD4 (BD Bioscience, RM4-5), APC Cy7 CD8 (Biolegend, 53-6,7), APC Ly6C (eBioscience, HK1,4), CD11b PE-Cy7 (BD Bioscience, M1/70) and 7-AAD (eBioscience). Cells were fixed with 4% paraformaldehyde, washed and analysed using a FACS Canto II. Neutrophils were identified as viable (CD45+, CD11b+, Ly-6G and -C+).

**RNA preparation and microarray profiling**

RNA was isolated from lung homogenates using the RNeasy mini kit (Qiagen, Venlo, The Netherlands) and processed exactly as described before [[5](#_ENREF_5)]. RNA integrity (RIN>7) was assessed by bioanalyzer (Agilent, Amstelveen, The Netherlands). Biotinylated cRNA was hybridized onto the Illumina MouseRef-8v2 Expression BeadChip. Samples were scanned using an Illumina iScan array scanner. Preparation of cRNA, chip hybridization, washing, staining and scanning were carried out at ServiceXS. The raw scan data were read using the beadarray package (version 1.12.1) [[6](#_ENREF_6)], available through Bioconductor [[7](#_ENREF_7)] in the R statistical environment (version 2.13.2; R Foundation for Statistical Computing). All non-normalized and neqc normalized [[8](#_ENREF_8)] data are available at the gene expression omnibus of NCBI (GEO) with accession number GSE53174. Differential gene expression analysis was performed by means of the limma package (version 3.8.3), which implements linear models for microarray data [[9](#_ENREF_9)]. P-values were obtained from moderated t statistics, which were then adjusted for multiple comparisons with Benjamini and Hochberg's method to control the false discovery rate. Bioinformatics analysis was performed on differentially expressed genes as defined by multiple-test corrected P <0.05 by means of the Ingenuity Pathway Analysis software (IPA, www.ingenuity.com). Fold changes and adjusted p-values per gene were imported into IPA. These analyses were performed using the IPA gene-only knowledgebase as reference set for p-value calculations and specifying “mouse” as species. All other parameters were maintained as default. Genes were stratified as high expression or low expression based on fold change (log2) of >0.2 or <-0.2, respectively. Significantly enriched canonical signalling pathways were demarcated by considering a Fisher’s test Bonferroni adjusted p-value <0.05.

**Cellular metabolism of alveolar macrophages**

Freshly harvested alveolar macrophages (via BAL as described above) from naïve control and antibiotic treated mice were seeded overnight at 5x10^4^ cells per well on Seahorse XF96 polystyrene tissue culture plates (Seahorse Bioscience, North Billerica, Massachusetts). The plate was incubated in unbuffered DMEM assay medium (Sigma-Aldrich, Zwijndrecht, The Netherlands) for 30 minutes in a non-CO2 incubator at 37°C before measuring in an XFe 96 extracellular flux analyzer (Seahorse Bioscience, North Billerica, Massachusetts). After baseline measurements, oxygen consumption rate (OCR) and extracellular acidification rate (ECAR, an indicator of lactate production) were measured over 4 min periods with a mixing of 2 or 3 min in each cycle, with four cycles in total. Oligomycin (which blocks mitochondrial complex V, where the electron transport chain is coupled to ATP synthesis), FCCP (carbonyl cyanide p-trifluoromethoxyphenylhydrazone, an uncoupling agent that allows maximum electron transport) and rotenone + antimycin A (which block complex I and III respectively, thereby blocking mitochondrial respiration) were sequentially added to each well as described before [[10](#_ENREF_10), [11](#_ENREF_11)]. Compounds were used at the following final concentrations: glucose (25mM), pyruvate (1 mM), glutamine (2 mM), Oligomycin (1,5 µM), FCCP (1,5 µM), Antimycin A (2,5 µM) and Rotenone (1,25 µM). DNA content of each well was measured using a CyQuant kit (Thermo Fisher scientific, Breda, The Netherlands) on a microplate reader. The data is represented as OCR or ECAR normalized to DNA content.

**Lung microbiota analysis**

Whole lungs were collected from uninfected control- and antibiotic pre-treated mice and immediately stored at -80 °C. DNA was isolated using a bead-beating protocol [[12](#_ENREF_12)] followed by DNA extraction using phenol and chloroform-isoamyl alcohol. The V1-V2 region of the bacterial 16S rRNA gene was amplified in a two-step protocol using universal primers 27F and 338R and sequenced on the Illumina MiSeq sequencing platform (Illumina, San Diego, CA, USA) as described before [[13](#_ENREF_13)] except for using 30 cycles in the first PCR and 10 cycles in the second PCR. Data was analyzed using NG-tax [[14](#_ENREF_14)]. Briefly, paired-end libraries were filtered to contain only read pairs with perfectly matching barcodes; these barcodes were used for demultiplexing. Operational Taxonomic Unit (OTU) picking was performed with an open reference approach and SILVA 16S rRNA gene reference database.

**References**

1. Weehuizen TA, Hommes TJ, Lankelma JM, de Jong HK, Roelofs JJ, de Vos AF, et al. Triggering Receptor Expressed on Myeloid Cells (TREM)-2 Impairs Host Defense in Experimental Melioidosis. PLoS neglected tropical diseases. 2016;10(6):e0004747. doi: 10.1371/journal.pntd.0004747. PubMed PMID: 27253382.

2. Wiersinga WJ, Wieland CW, Dessing MC, Chantratita N, Cheng AC, Limmathurotsakul D, et al. Toll-like receptor 2 impairs host defense in gram-negative sepsis caused by Burkholderia pseudomallei (Melioidosis). PLoS Med. 2007;4(7):e248. doi: 10.1371/journal.pmed.0040248. PubMed PMID: 17676990; PubMed Central PMCID: PMCPMC1950213.

3. de Stoppelaar SF, van 't Veer C, Claushuis TA, Albersen BJ, Roelofs JJ, van der Poll T. Thrombocytopenia impairs host defense in gram-negative pneumonia-derived sepsis in mice. Blood. 2014;124(25):3781-90. doi: 10.1182/blood-2014-05-573915. PubMed PMID: 25301709; PubMed Central PMCID: PMCPMC4263985.

4. Koh GC, Weehuizen TA, Breitbach K, Krause K, de Jong HK, Kager LM, et al. Glyburide reduces bacterial dissemination in a mouse model of melioidosis. PLoS neglected tropical diseases. 2013;7(10):e2500. Epub 2013/10/23. doi: 10.1371/journal.pntd.0002500. PubMed PMID: 24147174; PubMed Central PMCID: PMC3798430.

5. Schuijt TJ, Lankelma JM, Scicluna BP, de Sousa EMF, Roelofs JJ, de Boer JD, et al. The gut microbiota plays a protective role in the host defence against pneumococcal pneumonia. Gut. 2016;65(4):575-83. Epub 2015/10/30. doi: 10.1136/gutjnl-2015-309728. PubMed PMID: 26511795.

6. Dunning MJ, Smith ML, Ritchie ME, Tavare S. beadarray: R classes and methods for Illumina bead-based data. Bioinformatics. 2007;23(16):2183-4. doi: 10.1093/bioinformatics/btm311. PubMed PMID: 17586828.

7. Reimers M, Carey VJ. Bioconductor: an open source framework for bioinformatics and computational biology. Methods Enzymol. 2006;411:119-34. doi: 10.1016/S0076-6879(06)11008-3. PubMed PMID: 16939789.

8. Shi W, Oshlack A, Smyth GK. Optimizing the noise versus bias trade-off for Illumina whole genome expression BeadChips. Nucleic Acids Res. 2010;38(22):e204. doi: 10.1093/nar/gkq871. PubMed PMID: 20929874; PubMed Central PMCID: PMCPMC3001098.

9. Smyth GK. Linear models and empirical bayes methods for assessing differential expression in microarray experiments. Stat Appl Genet Mol Biol. 2004;3:Article3. doi: 10.2202/1544-6115.1027. PubMed PMID: 16646809.

10. Doddaballapur A, Michalik KM, Manavski Y, Lucas T, Houtkooper RH, You X, et al. Laminar shear stress inhibits endothelial cell metabolism via KLF2-mediated repression of PFKFB3. Arteriosclerosis, thrombosis, and vascular biology. 2015;35(1):137-45. doi: 10.1161/ATVBAHA.114.304277. PubMed PMID: 25359860.

11. Shen Y, Tian Y, Yang J, Shi X, Ouyang L, Gao J, et al. Dual effects of carnosine on energy metabolism of cultured cortical astrocytes under normal and ischemic conditions. Regul Pept. 2014;192-193:45-52. doi: 10.1016/j.regpep.2014.08.005. PubMed PMID: 25195162.

12. Salonen A, Nikkila J, Jalanka-Tuovinen J, Immonen O, Rajilic-Stojanovic M, Kekkonen RA, et al. Comparative analysis of fecal DNA extraction methods with phylogenetic microarray: effective recovery of bacterial and archaeal DNA using mechanical cell lysis. Journal of microbiological methods. 2010;81(2):127-34. Epub 2010/02/23. doi: 10.1016/j.mimet.2010.02.007. PubMed PMID: 20171997.

13. Lankelma JM, Cranendonk DR, Belzer C, de Vos AF, de Vos WM, van der Poll T, et al. Antibiotic-induced gut microbiota disruption during human endotoxemia: a randomised controlled study. Gut. 2016. doi: 10.1136/gutjnl-2016-312132. PubMed PMID: 27307305.

14. Ramiro-Garcia J, Hermes, G.D.A., Giatsis, C., Sipkema, D., Zoetendal, E.G., Schaap, P.J., Smidt, H. NG-Tax, a highly accurate and validated pipeline for analysis of 16S rRNA amplicons from complex biomes. F1000Research. 2016;5:1791.
